# Predicting Long-term Survival After Allogeneic Hematopoietic Cell Transplantation in Patients With Hematologic Malignancies: Machine Learning–Based Model Development and Validation

**DOI:** 10.2196/32313

**Published:** 2022-03-07

**Authors:** Eun-Ji Choi, Tae Joon Jun, Han-Seung Park, Jung-Hee Lee, Kyoo-Hyung Lee, Young-Hak Kim, Young-Shin Lee, Young-Ah Kang, Mijin Jeon, Hyeran Kang, Jimin Woo, Je-Hwan Lee

**Affiliations:** 1 Department of Hematology Asan Medical Center University of Ulsan College of Medicine Seoul Republic of Korea; 2 Big Data Research Center Asan Institute for Life Sciences Asan Medical Center Seoul Republic of Korea; 3 Division of Cardiology Asan Medical Center University of Ulsan College of Medicine Seoul Republic of Korea

**Keywords:** machine learning, hematopoietic cell transplantation, hematologic malignancies, prediction, survival, stem cell, transplant, malignancy, model, outcome, algorithm, bias, validation

## Abstract

**Background:**

Scoring systems developed for predicting survival after allogeneic hematopoietic cell transplantation (HCT) show suboptimal prediction power, and various factors affect posttransplantation outcomes.

**Objective:**

A prediction model using a machine learning–based algorithm can be an alternative for concurrently applying multiple variables and can reduce potential biases. In this regard, the aim of this study is to establish and validate a machine learning–based predictive model for survival after allogeneic HCT in patients with hematologic malignancies.

**Methods:**

Data from 1470 patients with hematologic malignancies who underwent allogeneic HCT between December 1993 and June 2020 at Asan Medical Center, Seoul, South Korea, were retrospectively analyzed. Using the gradient boosting machine algorithm, we evaluated a model predicting the 5-year posttransplantation survival through 10-fold cross-validation.

**Results:**

The prediction model showed good performance with a mean area under the receiver operating characteristic curve of 0.788 (SD 0.03). Furthermore, we developed a risk score predicting probabilities of posttransplantation survival in 294 randomly selected patients, and an agreement between the estimated predicted and observed risks of overall death, nonrelapse mortality, and relapse incidence was observed according to the risk score. Additionally, the calculated score demonstrated the possibility of predicting survival according to the different transplantation-related factors, with the visualization of the importance of each variable.

**Conclusions:**

We developed a machine learning–based model for predicting long-term survival after allogeneic HCT in patients with hematologic malignancies. Our model provides a method for making decisions regarding patient and donor candidates or selecting transplantation-related resources, such as conditioning regimens.

## Introduction

### Background

Allogeneic hematopoietic cell transplantation (HCT) is a potentially curative therapeutic option for patients with hematologic malignancies, which has been widely used. The increasing use of allogeneic HCT is attributable to multiple factors, including improved alternative donor availability, reduced-intensity conditioning regimens, advances in the prevention of transplantation-related toxicities, and an improvement in general supportive care. Despite these advances, allogeneic HCT remains associated with considerably high rates of complications, treatment-related mortality, and relapse [1]. To predict transplantation outcomes more accurately before making decisions regarding transplant eligibility, several prognostic scoring systems for survival after allogeneic HCT have been developed. These scores include the HCT-specific comorbidity index, the European Group for Blood and Marrow Transplantation (EBMT) risk score, and the Pretransplant Assessment of Mortality score, among others [2-5]. Most prognostic scoring systems were developed using parametric statistical methodologies, such as Cox proportional hazards models, for predicting the likelihood of survival for HCT recipients. The scoring systems’ variables mainly include recipient factors, such as age, comorbidities, performance status, time from diagnosis to HCT, and disease status. Furthermore, several donor factors are considered in the EBMT risk score, including donor type (human leukocyte antigen [HLA]: identical sibling or matched unrelated), sex match, and cytomegalovirus serostatus. However, the reported accuracy of these prediction models is suboptimal, where the area under the receiver operating characteristic (ROC) curve (AUC) ranges between 0.6 and 0.7 [6].

Recently, attempts to predict transplantation-related outcomes more accurately have been made in various clinical settings regarding early mortality [7], graft-versus-host disease (GVHD) [8], or relapse using deep learning–based prediction models [9]. The Acute Leukemia (AL)–EBMT score was developed using a data mining–based approach to predict 100-day mortality after allogeneic HCT [7]. Another study of a machine learning algorithm predicting the incidence of acute GVHD using Japanese registry data has demonstrated that the calculated scores were associated with clear stratification of acute GVHD, whereas lower scores were associated with a low incidence of acute GVHD [8]. In one study, a machine learning–based model was developed to predict the 1-year relapse rate after allogeneic HCT in patients with AL [9]. Although the patient population, endpoints, and criteria for variable selection were different between studies, the performances were similar and seemed slightly better than the previously reported transplantation outcome prediction scores.

The survival following allogeneic HCT, however, can vary depending on multiple variables, such as disease relapse and transplantation-related complications, including GVHD, engraftment failure, or infection, which can lead to increased nonrelapse mortality (NRM). Furthermore, these HCT complications are associated with several variables, including donor-related or recipient-related factors, donor-recipient relationship, and conditioning, among others.

### Objective

We hypothesized that the selection of variables using a machine learning–based approach and the establishment of a prediction model by applying those variables will improve the performance of the model and avoid unexpected biases. Additionally, we assumed that the established prediction algorithm will help choose better transplantation-related factors or donors to improve post-HCT outcomes. In this study, we developed a model for predicting the long-term survival of patients with hematologic malignancies after allogeneic HCT based on selected variables using a machine learning algorithm, and we validated the model’s accuracy in a validation set. Then, we implemented an algorithm to select more appropriate transplantation-related factors using the established prediction model.

## Methods

### Patient Population and Study Outcomes

Data on 1470 adult patients (≥15 years old) with hematologic malignancies who underwent allogeneic HCT between December 1993 and December 2015 at Asan Medical Center, Seoul, South Korea, were obtained for developing the machine learning–based prediction model. To predict long-term survival after allogeneic HCT, we included patients who survived more than 5 years and who died within 5 years after transplantation. As the data cutoff date was December 2020, we only included patients who underwent allogeneic HCT before December 2015 to ensure that the follow-up duration of each patient could be at least 5 years. Then, 229 variables, including recipient and donor characteristics, disease features, HLA types, graft information, administered medications for conditioning, GVHD prophylaxis, supportive care, and other laboratory data, were collected for analysis.

The primary objective of the study was to predict the 5-year overall survival (OS) after allogeneic HCT, and the secondary objectives include determining the NRM, cumulative incidence of relapse (CIR), and 100-day OS. All censored data were calculated from the date of the transplantation.

### Ethics Approval

The Institutional Review Board of Asan Medical Center approved the protocols of this study (2021-1003), which was conducted according to the 2008 Declaration of Helsinki.

### Selection of a Predicting Model

The patients were classified into two groups, those who survived more than 5 years and those who died within 5 years. In the learning process, the former group was labeled 0 and the latter was labeled 1. Therefore, the closer the predicted value to 1, the higher the probability of death within 5 years. The aforementioned predictive factors were classified into categorical or noncategorical variables and used for developing 5 prediction models. The performance for predicting survival after allogeneic HCT was tested using the following 5 machine learning algorithms: gradient boosting machine (GBM), random forest, deep neural network, logistic regression, and adaptive boosting (AdaBoost). Each algorithm was tested using the same training set which was randomly divided (1176/1470, 79.59% of the total number of patients in the training set). The AUCs of the algorithms are shown in Multimedia Appendix 1. Of the 5 algorithms, GBM showed the highest AUC (0.75) compared with random forest (0.74), deep neural network (0.65), logistic regression (0.70), and AdaBoost (0.72). Therefore, the selection of relevant variables and the development of the final model were performed using GBM. GBM is an ensemble method that combines several weak classifiers, such as trees. The goal of GBM is to focus and place the weights on incorrectly predicted results through gradient descent [10]. While GBM is training, the initial tree trains the data set and assigns weights to incorrectly predicted records with errors, and the next tree from the same model learns the weighted data set and repeats the process of assigning weights.

### Explainable Individualized Survival Prediction

We provided an explainable individualized survival prediction using Shapley values to quantify the probability of surviving for each patient by predicting the OS after allogeneic HCT. A Shapley value is calculated as the average change according to the presence or absence of a single feature over all possible combinations of features [11]. Given a survival prediction model, f(x), we can compute the Shapley values using the following equation:




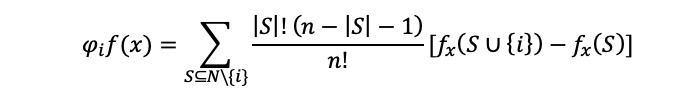




where *n* is the total number of features, and the sum extends over all subsets *S* of *N* not containing feature *i*. In a recent study, a unified framework called Shapley Additive Explanations (SHAP) was released for explainable machine learning models using Shapley values [10]. In this study, the survival model also provides a description of patient-specific survival prediction using SHAP.

### Other Statistical Analyses

Categorical variables were compared using the chi-square test or Fisher exact test, and continuous variables were compared using the Mann-Whitney *U*-test or Student *t* test, as appropriate. The OS was calculated using the Kaplan-Meier method, and the resulting survival curves were compared using the log-rank test. NRM and CIR were evaluated using a cumulative incidence function regarding competing risks and compared using the method of Gray in R, version 3.6.3 (R Foundation for Statistical Computing). All statistical analyses were conducted using SPSS, version 24 (IBM Corp), and graphs were generated using GraphPad Prism, version 9.1.2 (GraphPad Software Inc). In all analyses, *P* values were two-tailed, and those less than .05 were used to denote statistical significance.

## Results

### Patient and Donor Characteristics

The characteristics of the patients and donors included in the study are shown in Table 1. Between December 2009 and December 2015, 1470 patients underwent allogeneic HCT for hematologic malignancies, including acute myeloid leukemia (n=783), acute lymphoblastic leukemia (ALL; n=306), myelodysplastic syndrome (MDS; n=188), chronic myeloid leukemia (n=92), non-Hodgkin/Hodgkin lymphoma (n=56), BCR-ABL1–negative myeloproliferative neoplasm (MPN; n=16), MDS/MPN (n=6), and multiple myeloma (n=13). Approximately two-thirds of the patients (n=995) received peripheral blood as a graft source, and one patient who received cord blood as a graft source was included. Reduced-intensity conditioning and myeloablative conditioning were used in 934 (63.5%) and 536 (36.5%) of the 1470 patients, respectively. Antithymocyte globulin was used in 903 (61.4%) of the 1470 patients as GVHD prophylaxis.

During the median follow-up duration of 8 years (95% CI 7.8-8.3 years), the estimated 5-year OS of all patients was 46.2%. The 2-year incidence of NRM and CIR was 17.7% and 33.3%, respectively.

**Table 1:** Patient and donor characteristics.

Variable		Value
Patients, N		1470
Interval between diagnosis to HCT^a^ in months, median (95% CI)		5.7 (0-268)
**Recipient sex, n (%)**		
	Male	833 (56.7)
	Female	637 (43.3)
**Donor sex, n (%)**		
	Male	977 (66.5)
	Female	493 (33.5)
Recipient age in years, median (range)		41 (15-75)
Donor age in years, median (range)		34 (0-70)
**Donor-recipient sex, n (%)**		
	Male to male	551 (37.5)
	Female to male	280 (19)
	Male to female	424 (28.8)
	Female to female	213 (14.5)
**Recipient disease, n (%)**		
	AML^b^	783 (66.9)
	MDS^c^	188 (16.1)
	ALL^d^	306 (26.2)
	Lymphoma	56 (4.8)
	MM^e^	13 (1.1)
	CML^f^	92 (7.9)
	MPN^g^	16 (1.4)
	MDS-MPN	16 (1.4)
HCT-CI^h^ score, median (range)		3 (0-8)
**Disease risk, n (%)**		
	Standard risk^i^	830 (56.5)
	High risk	640 (43.5)
**Donor type, n (%)**		
	Matched sibling	591 (40.2)
	Unrelated	387 (26.4)
	Haploidentical familial	491 (33.4)
	Cord blood	1 (0.1)
**Graft source, n (%)**		
	Bone marrow	472 (32.1)
	Peripheral blood	997 (67.8)
	Cord blood	1 (0.1)
**Conditioning intensity, n (%)**		
	Myeloablative	536 (36.5)
	Reduced intensity	934 (63.5)
Treated with antithymocyte globulin to prevent GVHD^j^, n (%)		903 (61.4)

^a^HCT: hematopoietic cell transplantation.

^b^AML: acute myeloid leukemia.

^c^MDS: myelodysplastic syndrome.

^d^ALL: acute lymphoblastic leukemia.

^e^MM: multiple myeloma.

^f^CML: chronic myeloid leukemia.

^g^MPN: myeloproliferative neoplasm.

^h^HCT-CI: hematopoietic cell transplantation–specific comorbidity index.

^i^The standard-risk group is defined as follows: patients with acute leukemia in the first remission (except by salvage chemotherapy), CML in the chronic phase, drug-sensitive lymphoma/MM, or MDS with bone marrow blasts ≤5% at HCT.

^j^GVHD: graft-versus-host disease.

### Development of the Prediction Model

After deciding on GBM as the prediction algorithm, the variables used for model development were selected using the recursive feature elimination (RFE) method. RFE is one of the widely used feature selection methods that provide a rank to each variable according to feature importance in predicting the target variable and help select a minimum specified number of variables showing good performance in a model [12]. Using the RFE algorithm, we selected 45 relevant variables for developing the prediction model (see Multimedia Appendix 2 and Textbox 1). In the case of HLA type, each allele of recipients and donors was regarded as an independent variable.

Selected variables for the prediction model. AML: acute myeloid leukemia. WBC: white blood cell. HLA: human leukocyte antigen. RBC: red blood cell. CMV: cytomegalovirus. HCT-CI: hematopoietic cell transplantation–specific comorbidity index. *The standard-risk group is defined as follows: patients with acute leukemia in the first remission (except by salvage chemotherapy), CML in the chronic phase, drug-sensitive lymphoma/MM, or MDS with bone marrow blasts ≤5% at HCT.

**Variables**

- Diagnosis and disease (eg, AML first complete remission)
- Disease risk*
- WBC count at diagnosis
- Extramedullary disease at diagnosis
- Extramedullary disease at HCT
- Karyotype at diagnosis
- Karyotype at HCT
- CMV serostatus of recipient
- CMV serostatus of donor
- Hepatic score of HCT-CI
- Total score of HCT-CI
- Conditioning regimen
- Donor type
- Recipient HLA type: A, B, C, DR, and DQ
- Donor HLA type: A, B, C, DR, and DQ
- RBC transfusion before HCT
- Platelet transfusion before HCT

### Final Performance of the Prediction Model

**Figure 1:**
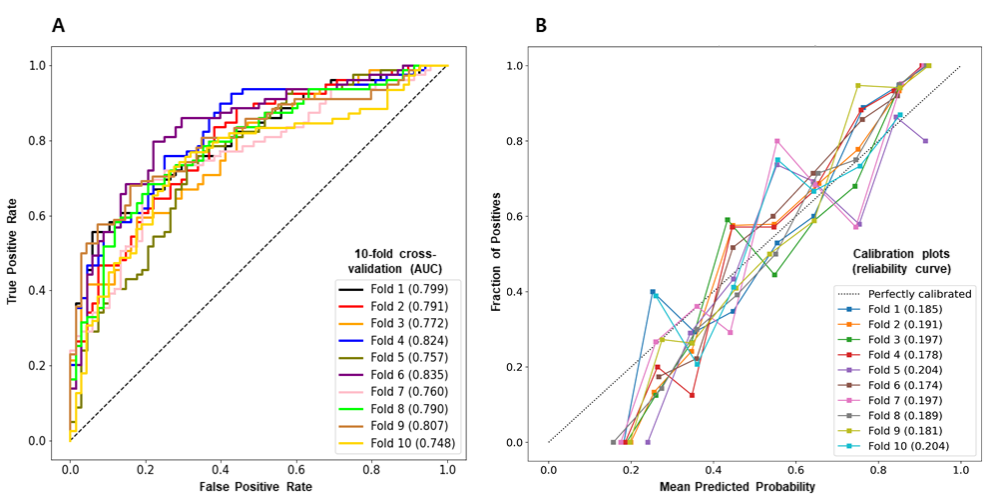
The final performance of the prediction model. Panel A shows the area under the receiver operating characteristic curve. Panel B shows the calibration plot.

The performance of the prediction model using GBM and selected variables in 294 patients is depicted in Figure 1A. The AUC and prediction accuracy of the final model were 0.788 and 0.712, respectively. Figure 1B shows a calibration plot with the Brier score of the model demonstrating agreement between the estimated predicted risk and observed risk of death in the validation cohort. The algorithm was trained and evaluated using 10-fold cross-validation in the total patient cohort, where the predictive power of the model demonstrated a generalized performance with a similar accuracy.

**Figure 2:**
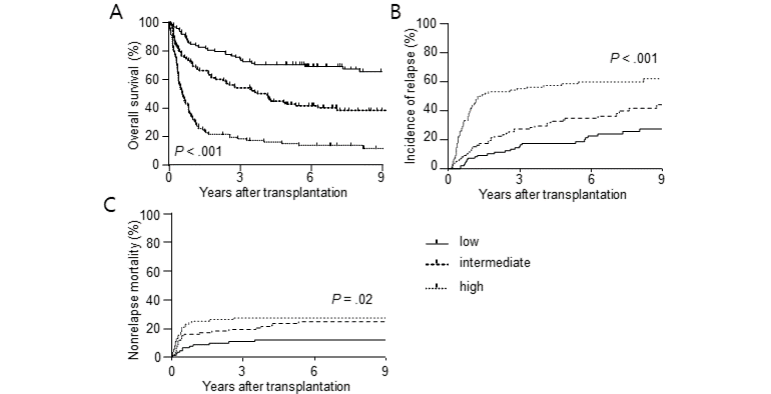
Different post-transplantation outcomes of the patients of validation set according to the prediction score (A) Overall survival (B) relapse (C) non-relapse mortality.

Because we classified patients who died within 5 years as 1, the closer the predicted value of the GBM model to 1, the higher the risk of death. The optimal threshold for determining whether the risk score is positive or negative is calculated using the Youden J statistic along with the ROC curve. From the prediction model, the threshold is 0.5533, and if the risk score is greater than that, the model estimates that the patient will die within 5 years. The predicting probability of the risk score of each patient was tested in a randomly selected patient cohort, which corresponds to 20% of all patients (294/1470) to reduce the probable bias from choosing one of 10 produced models. Figure 2A shows the estimated post-transplantation OS of the patients according to the risk score, which was equally divided by the absolute score values. The estimated 5-year OS was 70.3% in the low-risk group, 42.6% in the intermediate-risk group, and 14.9% in the high-risk group (*P*<.001).

### Prediction of NRM and Relapse

To assess whether the risk score can also predict NRM and relapse after HCT, we analyzed the incidence of NRM and relapse using 3 risk groups. High-risk scores were significantly associated with both higher CIR (*P*<.001) and higher NRM (*P*=.02) (Figure 2B and 2C). The estimated 2-year CIR was 11.3% in the low-risk group, 22.4% in the intermediate-risk group, and 53.1% in the high-risk group. The 2-year NRM was 9.3% in the low-risk group, 17.3% in the intermediate-risk group, and 25% in the high-risk group.

### Application of the Algorithm for Donor Selection

**Figure 3:**
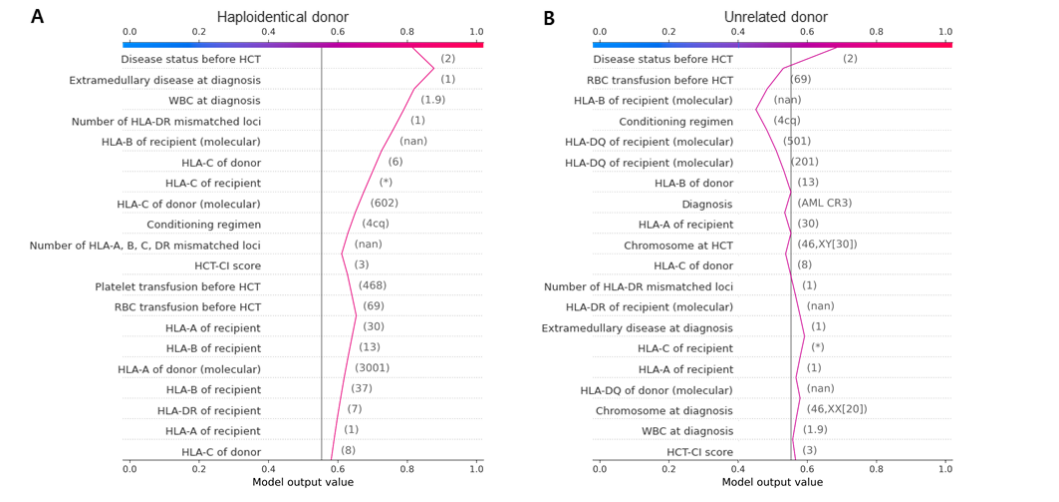
Survival difference of the patients of validation set according to the prediction score.

We assumed that the prediction score for each patient can be applied in selecting the most appropriate donor when there are multiple donor candidates. For example, the prediction score can help physicians select the donor between a younger HLA-haploidentical individual and an older matched sibling. To verify this, we calculated the scores using Shapley values through which the importance of each variable can be visualized using a specific value. We simulated a real case of a patient with ALL in the first CR who has the following 2 donor candidates: one is a 48-year-old HLA-haploidentical familial female individual, and the other is a 43-year-old locus-mismatched unrelated male individual. A total of 2 prediction scores were calculated using data derived from each donor showing different values (Figure 3). Among the variables, the pretransplantation disease status appeared to be the most important factor in calculating each score. According to our prediction model, the donor in Figure 3B (unrelated donor) could be preferred because the score is lower than the donor in Figure 3A (haploidentical donor).

## Discussion

### Principal Findings

Long-term survival after allogeneic HCT in patients with hematologic malignancies is affected by multiple factors but mainly depends on disease relapse and NRM. Multiple variables, including disease status, genetic risk, conditioning regimen, comorbidities, degree of HLA matching, and patient and donor ages, are associated with disease relapse, GVHD, engraftment, or treatment-related toxicities, and these outcomes are closely and mutually related to survival after transplantation. However, traditional statistical methods are unsuitable for analysis considering the interactions between variables or their differences according to the specific values of each factor, such as the relationship between the HLA allele of the patient and donor. In this regard, prediction models based on machine learning algorithms can be an effective alternative for predicting posttransplantation outcomes and can provide guidance for selecting appropriate patients, donors, or resources [13].

We developed a prediction model and risk score using GBM and selected variables based on machine learning for long-term survival after allogeneic HCT. Our model demonstrated an AUC of 0.788, which showed better performance in predicting posttransplantation outcomes than previously reported machine learning–based models. Shouval et al [7] have reported the AL-EBMT model predicting 100-day mortality after allogeneic HCT showing an AUC of 0.701, which was significantly better than that of the EBMT score (AUC, 0.646). A study on Japanese individuals who underwent HCT has developed a machine learning–based prediction algorithm of acute GVHD, and the AUC of the model was 0.62 [8]. Another prediction algorithm developed by Fuse et al [9] has shown an AUC of 0.667 for predicting relapse within 1 year after transplantation. Most models were developed by applying the alternating decision tree algorithm, and the variables were selected by researchers. In this study, the model was developed using variables derived from the GBM algorithm and using the RFE method, instead of using preselected variables based on the opinion of the researchers or conventional statistical analysis. Through RFE, we extracted the minimum required features where the performance of the predicting model does not deteriorate. This is an important difference from the existing literature that applied machine learning algorithms using clinically selected variables. In contrast, we first built a full model using all possible variables and then gradually removed features that had little effect on survival prediction. Those differences might contribute to the higher AUC of our prediction model by reducing biases in selecting variables and augmenting possible correlations between each factor. Interestingly, the selected variables for our prediction model include each HLA allele type of recipients and donors. Because we used the raw values of each HLA allele of both recipients and donors rather than calculating the degree of mismatch, direction of mismatch, or allele types, our approach integrated the interactions between alleles affecting survival.

To apply the prediction model to patients planning for allogeneic HCT in practice, a specific tool for comparing the expected outcomes according to multiple different factors is required. We provided a prediction score to quantify the probability of survival, which showed good concordance of the observed and estimated survival after HCT. Additionally, SHAP visualizes the importance of each factor (Figure 3), which allows for the prioritization of more appropriate transplantation-related resources. The most remarkable aspect of our model is that the importance of each factor can be quantified and visualized so that physicians can use the algorithm when planning allogeneic HCT to select factors, such as donor or conditioning regimen, that are expected to achieve better survival.

The limitations of this study include the relatively small number of patients used for establishing the algorithm-based prediction model. Although the model showed consistency using 10-fold cross-validation in the validation cohort, a larger patient cohort is considered more helpful in verifying the performance of the algorithm. Further external validation using data from a greater number of patients is warranted. Second, the retrospective nature of the study may have resulted in selection and measurement biases. However, we included all patients with hematologic malignancies who underwent allogeneic HCT during a certain period of time to reflect real-world practice.

### Conclusions

Here, we present a machine learning–based algorithm and prediction score for quantifying the probability of long-term survival after allogeneic HCT in patients with hematologic malignancies. The prediction score showed a moderate negative correlation with long-term survival, NRM, and relapse after transplantation. Our prediction model provides a personalized method for selecting more appropriate transplantation-related factors and patient or donor candidates for allogeneic HCT.

## Appendix

Multimedia Appendix 1 The AUC of each tested algorithm. cb, CatBoost; rf, random forest; fnn, feedforward neural network; log, logistic regression; ada, AdaBoost.

Multimedia Appendix 2 Recursive feature elimination showing the AUC (area under the curve) according to the number of selected features.

## Abbreviations

ALL: acute lymphoblastic leukemia
AUC: area under the ROC curve
CIR: cumulative incidence of relapse
GBM: gradient boosting machine
GVHD: graft-versus-host disease
HCT: hematopoietic cell transplantation
HLA: human leukocyte antigen
MDS: myelodysplastic syndrome
MPN: myeloproliferative neoplasm
NRM: nonrelapse mortality
OS: overall survival
RFE: recursive feature elimination
ROC: receiver operating characteristic
SHAP: Shapley Additive Explanations
