# Supplementary figures and images for: Predicting Long-term Survival After Allogeneic Hematopoietic Cell Transplantation in Patients With Hematologic Malignancies: Machine Learning–Based Model Development and Validation
Source: JMIR Med Inform. 2022 Mar 7;10(3):e32313. doi: 10.2196/32313 (PMC8938832; doi:10.2196/32313)

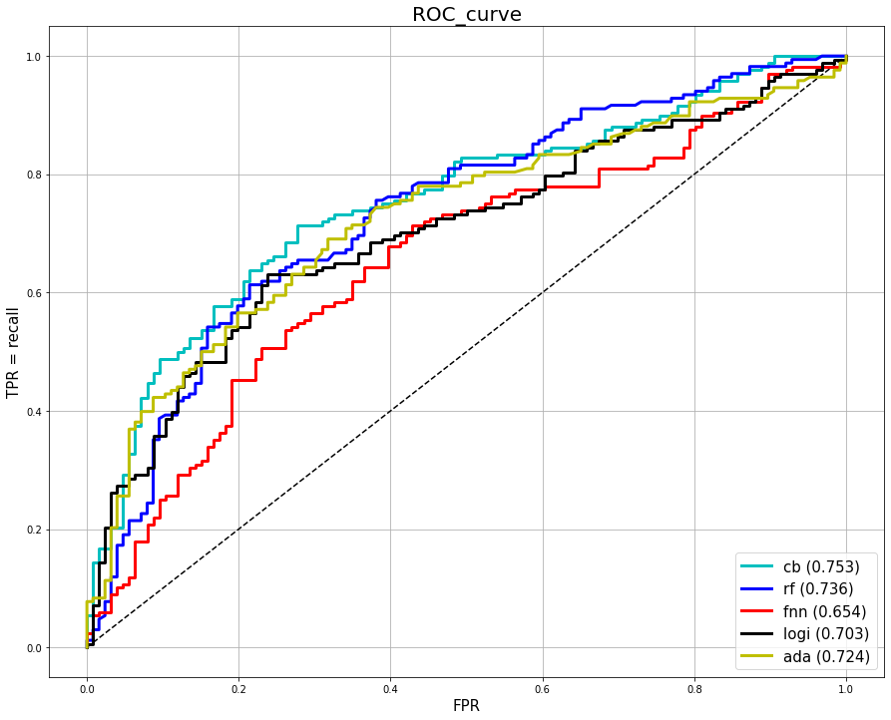

Supplement: Multimedia Appendix 1 [file medinform_v10i3e32313_app1.png]

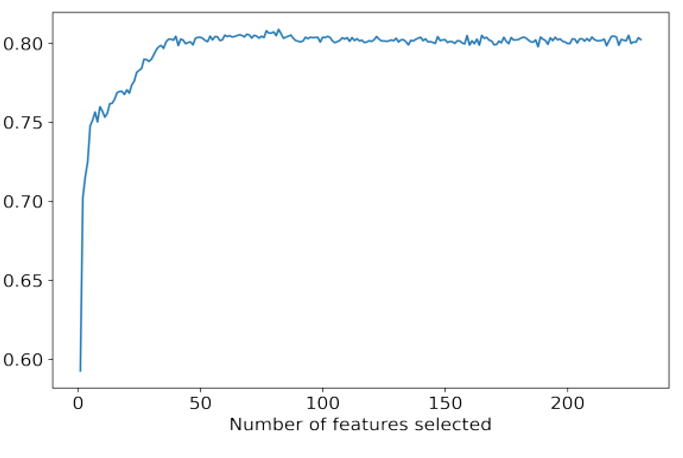

Supplement: Multimedia Appendix 2 [file medinform_v10i3e32313_app2.png]
